# Effect of graded nursing based on the Glasgow-Blatchford score in liver cirrhosis patients complicated with acute upper gastrointestinal bleeding

**DOI:** 10.3389/fmed.2025.1679939

**Published:** 2025-11-27

**Authors:** Mingshu Liu, Haifang Wang, Fei Shao, Yanfang Xu, Jing Huang, Huijie Zhang, Yaru Han

**Affiliations:** 1 Department of Hepatology Center, The First Hospital of Hebei Medical University, Shijiazhuang, Hebei, China; 2 Department of Infectious Diseases, The First Hospital of Hebei Medical University, Shijiazhuang, Heibei, China; 3 Department of General Surgery, The First Hospital of Hebei Medical University, Shijiazhuang, Heibei, China

**Keywords:** liver cirrhosis, acute upper gastrointestinal bleeding, Glasgow-Blatchford score, graded nursing, re-bleeding

## Abstract

**Aim:**

To study the effect of graded nursing based on the Glasgow-Blatchford score in liver cirrhosis patients complicated with acute upper gastrointestinal bleeding (AUGIB).

**Methods:**

From January 2022 to December 2024, eighty patients with liver cirrhosis complicated with AUGIB treated in our hospital were chosen and separated into control group and study group. The control group received routine nursing, and the study group received graded nursing based on the Glasgow-Blatchford score. The hemostatic time and hospital stay, number of patients with re-bleeding and death, Glasgow-Blatchford score, incidence of complications, psychological states, self-care ability, quality of life and nursing satisfaction were compared between the two groups.

**Results:**

Compared to the control group, the study group had shorter hemostatic time and hospital stay, lower rate of re-bleeding and death, lower incidence of complications and higher nursing satisfaction (*P* < 0.05 and *P* < 0.01). At discharge, the Glasgow-Blatchford score was declined, the self-rating anxiety scale (SAS) and self-rating depression scale (SDS) scores were declined, the exercise of self-care agency (ESCA) scores were elevated and the Short Form-36 (SF-36) scores were elevated in both groups (*P* < 0.05). Compared to the control group, the study group had lower Glasgow-Blatchford score, lower SAS and SDS scores, higher ESCA scores and higher SF-36 scores at discharge (*P* < 0.05).

**Conclusion:**

Graded nursing based on the Glasgow-Blatchford score can reduce the re-bleeding rate and incidence of adverse reactions, alleviate the anxiety and depression and enhance the self-care ability, quality of life and nursing satisfaction of patients with liver cirrhosis combined with AUGIB.

## Introduction

Liver cirrhosis is a diffuse liver damage caused by one or more factors acting repeatedly over a long period of time (1). In the early stage, the liver may have a strong compensatory function and show no obvious clinical manifestations; while in the later stage, liver function damage and portal hypertension may occur, which may lead to acute upper gastrointestinal bleeding (AUGIB) (2). AUGIB mainly refers to acute bleeding caused by lesions above the Treitz ligament in the digestive tract, including pancreatic duct, bile duct and anastomotic diseases caused by gastrojejunostomy (3). The clinical manifestations of AUGIB include hematemesis, black stool, and bloody stool (4). When the blood loss within a short period exceeds 1000 mL or exceeds more than 20% of the circulating blood volume, it may cause peripheral circulation disorders, and in severe cases, it may even endanger life (5). It is estimated that 48–165 out of every 100,000 adults will experience AUGIB each year (6). Patients with AUGIB have serious adverse events, including re-bleeding (5%–15%) and death (1%–13%) (7). Endoscopic examination can stop bleeding in some patients at an early stage of their condition, but up to 15% of patients will still bleed again after endoscopic treatment, which may lead to the death of some patients (8). Initial condition assessment helps to formulate subsequent treatment plans, and grading care based on risk stratification can significantly improve the prognosis of patients (9). Therefore, accurate early risk classification for AUGIB patients is the primary issue that clinicians need to consider (10).

The Glasgow-Blatchford score is a commonly used scoring system for evaluating AUGIB (11). This score was proposed by Oliver Blatchford in 2000 and consists of systolic blood pressure, hemoglobin, blood urea nitrogen, and other clinical manifestations, used to predict whether hospitalization intervention or death risk is required after AUGIB (12). In this study, the severity of the condition of patients with liver cirrhosis combined with AUGIB was evaluated, and graded nursing was implemented based on the Glasgow-Blatchford score, aiming to provide a new reference for the nursing of such patients.

## Data and methods

### General data

From January 2022 to December 2024, eighty patients with liver cirrhosis complicated with AUGIB treated in our hospital were chosen and separated into control group and study group based on the random number table method, and each group had 40 cases. After detailed recording and verification, no cases of sample loss, incomplete questionnaires or loss of participants during the follow-up were observed throughout the study period. Inclusion criteria: (1) Patients met the diagnostic criteria for liver cirrhosis in the 2019 Guidelines for the Diagnosis and Treatment of Cirrhosis (13); (2) Combined with clinical symptoms, signs and endoscopic results, AUGIB was confirmed; (3) The patient was conscious and could communicate with the medical staff normally. Exclusion criteria: (1) Upper gastrointestinal bleeding not caused by liver cirrhosis; (2) Patients had lower gastrointestinal bleeding; (3) Patients had serious diseases such as malignant tumors; (4) Patients who were unable to communicate normally with medical staff due to aphasia or depression. Patients or their family members voluntarily signed informed consent.

#### Recruitment of nursing and medical staff

All the nurses involved in the care of patients in this study were selected through our hospital’s strict recruitment process. Nurses are required to have a college degree or above in nursing, hold a valid nurse practice qualification certificate, and have at least 1 year of clinical nursing experience. Nurses with more than 1 year of working experience in the Department of Gastroenterology or hepatology was given priority. Such standards were designed to ensure that the nurses participating in the research had solid professional basic knowledge, proficient clinical operation skills, and experience in dealing with nursing issues related to liver diseases.

The medical staff involved in the diagnosis and treatment of patients in this study mainly included doctors with the title of attending physician or above. When recruiting, doctors were required to have a bachelor’s degree or above in medicine, hold the corresponding physician’s practice qualification certificate and have a professional title of attending physician or above. They should have at least 5 years of clinical experience in the field of liver diseases or gastroenterology, and those who had participated in relevant liver disease research projects or clinical research were given priority.

After the recruitment was completed, a qualification review team composed of the hospital’s human resources department, nursing department and medical affairs department conducted a strict review of the qualifications of the recruited personnel. The review content included the authenticity and validity of academic certificates, professional qualification certificates, and professional title certificates, as well as the verification of work experience. Only those who passed the review were eligible to participate in the nursing and medical treatment work related to this research.

#### Training program of nurses

A 2-weeks centralized training was conducted for the selected nurses. The training content covered the pathophysiological knowledge, common symptoms and key points of nursing for liver cirrhosis complicated with AUGIB, the specific content, scoring method and clinical significance of the Glasgow-Blatchford score, the operation process of graded nursing, the key points and precautions of nursing for patients of different grades, first aid skills training (including cardiopulmonary resuscitation, hemostasis and bandaging), and venipuncture, doctor-patient communication skills.

The training method adopted a combination of theoretical instruction and practical operation. Theoretical lectures were conducted by inviting liver disease experts and senior nursing lecturers from the hospital. Through multimedia courseware, case analysis and other forms, the understanding and mastery of knowledge by nurses were deepened. The practical operation was carried out in the simulated ward, with experienced nurses demonstrating and guiding, allowing the nurses to practice the actual operation and correct the problems in the operation in a timely manner.

After the training, theoretical and operational assessments were conducted for the nursing staff. The theoretical assessment was conducted in a closed-book format, with a full score of 100 points. A score of 80 points or above was considered qualified. The operation assessment was conducted by an assessment team who observed the operation process of the nurse on-site. Scores were given based on the operation norms and proficiency, with a full score of 100 points. A score of 85 points or above was considered qualified. Only personnel who passed both theoretical and operational assessments were eligible to participate in the patient care work of this study.

#### Training program of medical staff

A 1-week specialized training was conducted for the selected medical staff. The training focused on the latest diagnosis and treatment progress of liver cirrhosis complicated with AUGIB, the application of Glasgow-Blatchford score in disease assessment and prognosis judgment, the key points of collaboration with the nursing team under the concept of graded nursing, and the standardized requirements for data collection and recording in clinical research.

The training methods mainly consisted of special lectures and case discussions. We invited renowned experts in the field of liver diseases in China to give special lectures, sharing cutting-edge knowledge and clinical experience. We organized case discussion meetings, selected typical cases of liver cirrhosis complicated with AUGIB, had medical staff analyze and discuss them, propose treatment plans, and have experts provide comments and guidance to improve the clinical thinking ability and decision-making level of medical staff.

After the training, medical staff were evaluated through case analysis assessment and clinical operation assessment. Case analysis assessment required medical staff to formulate detailed treatment plans based on the given case information and provide written explanations. The expert group scored the plans, with a full score of 100 points, and a score of 80 points or above was considered qualified. The clinical operation assessment mainly examined the operational skills of medical staff in emergency treatment, endoscopic examination and treatment. It was also scored by an expert group, with a full score of 100 points, and a score of 85 points or above was considered qualified. Only those who passed the assessment were eligible to participate in the patient diagnosis and treatment work of this study.

### Methods

The control group received routine nursing, including routine emergency nursing, drug hemostasis or endoscopic hemostasis, rapid opening of venous channels, blood volume supplementation, close observation of vital signs and appropriate psychological nursing.

The study group received graded nursing based on the Glasgow-Blatchford score, including:

(1) According to systolic blood pressure (SBP), blood urea nitrogen, hemoglobin (Hb), pulse, black stool and other indicators and symptoms of patients, the bleeding risk was assessed via the Glasgow-Blatchford score system. The sum of all scores was used as the scoring result, and the bleeding risk of patients was divided into three levels: low, medium and high, and different levels of targeted nursing were implemented. Classification criteria: ≤6 points were defined as low risk, 7–9 points was defined as medium risk, ≥10 points were defined as high risk.

(2) Implementation of graded nursing: ➀ Low-risk group: Patients in this group had a low-risk degree, and the probability of re-bleeding after bleeding stops was small, so they were arranged in ordinary wards, but they still needed to strengthen disease observation to prevent disease aggravation. Before initiating educational intervention for patients in the low-risk group, there were clear and strict standards. The vital signs of patients remained stable, with systolic blood pressure maintained at 90–140 mmHg, heart rate at 60–100 beats per minute, and respiratory rate at 12–20 beats per minute. Moreover, through routine monitoring and simple treatment measures, a stable state could be continuously maintained. In addition, the patient’s state of consciousness was clear, capable of accurately understanding the instructions and explanations given by medical staff, and possessed good cognitive and communication skills to ensure full participation and understanding of the content of health education. Only patients who simultaneously met the Glasgow-Blatchford low-risk score and all the above clinical conditions were selected to receive the specific early admission health education intervention in this study. During hospitalization, the health education courses for patients in the low-risk group were arranged as follows: the first centralized education was conducted at the beginning of admission, and then intensive education was carried out every other day until the patients were discharged. The health education courses for patients in the low-risk group upon admission were mainly conducted by the nurses. All the nurses had received professional training and possessed rich knowledge of nursing for liver cirrhosis complicated with AUGIB as well as health education skills. Before each health education session, the nurse, based on the established educational syllabus and in combination with the individual circumstances of the patient, such as age, educational level, and understanding of the disease, carefully prepared educational materials like picture, video, and PPT. During the health education process, the nurse explained to the patients in plain and understandable language the influencing factors that induce AUGIB, and patiently answered the questions raised by the patients to ensure that they could fully understand the educational content. In addition, the nurse also regularly communicated and discussed with other members of the nursing team, shared experiences and problems in the educational process, and continuously optimized the educational methods and effects. In the early stage of admission, patients were given health education in the form of pictures, videos, and PPT, to inform them of the influencing factors that cause AUGIB. The total duration was controlled within 30–40 min. Among them, the display time for pictures was 10–15 min, the video playback time was 10–15 min, and the PPT presentation time was 10–15 min. According to the evaluation results, targeted treatment and nursing of hypertension, cirrhosis and other diseases were implemented. On this basis, the patient’s self-management ability was trained to promote them to develop good habits, strict control of diet and rest time, and fundamentally prevent disease aggravation. In this study, the average hospital stay for patients at low risk was 7 days. During the hospitalization period, from the initial centralized education to the subsequent daily reinforcement education arrangements, all could be completed in an orderly manner before the patients were discharged, ensuring that they received complete and systematic health education and intervention. Before discharge, the nurse established a health file for the patient, recorded the patient’s contact information and condition. After the patient was discharged, in order to ensure the effectiveness of continuous education, the nursing team formulated a follow-up plan based on the individual condition of the patient. During the first month after discharge, a telephone follow-up was conducted once a week. Subsequently, it was adjusted to once every 2 weeks or once a month, depending on the recovery status. The follow-up contents covered aspects such as disease monitoring, medication guidance, diet and lifestyle adjustment, in order to achieve continuous education management. The nursing of low-risk patients was mainly arranged by nurses with low seniority (These nurses have at least 1 year but no more than 3 years of clinical nursing experience). To assess the patients’ understanding after education, this study adopted the interview assessment method: During the patient’s hospitalization and before discharge, nurses conducted one-on-one interviews with the patient, inquiring about the patient’s understanding and application of disease knowledge and self-management methods. Based on the patient’s responses, a comprehensive assessment was made to determine whether the patient truly understood the educational content and could apply it reasonably in daily life. ➁ Medium risk group: Patients in this group had a higher risk degree and were prone to re-bleeding. Therefore, the level of nursing was strengthened and they were arranged in the emergency room or a ward closer to the nurse station. Nurses carefully observed the symptoms of upper gastrointestinal bleeding, prepared the rescue supplies at any time, and performed emergency rescue on the patients when their condition changed. At least two nurses were on night duty in the ward of patients in the medium-risk group, closely monitoring the patients’ vital signs such as breathing, blood pressure, and heart rate. If bleeding occurred at night, nurses immediately notified the doctor on duty and cooperated with the doctor for emergency treatment. Due to the severity of the patients’ conditions and the possibility that they may develop depression due to concerns about the disease, the nurses considered the patients’ perspectives fully, established a good relationship with the patients, gained their trust, and provided psychological care. At the same time, nurses paid attention to strengthen the basic nursing of patients to prevent the occurrence of infection after bleeding. When the patients were discharged, their addresses and contact information were registered, and follow-up calls were made every 2 weeks and home visits every 2 months to observe the patients’ recovery and precautions for family care, and provide relevant education. The patients in the medium-risk group were cared for by nurses with a junior or higher professional title and at least 3 years of working experience. ➂ High risk group: The patients in the high-risk group were in a very critical condition and their condition changed rapidly. These patients were placed in the emergency room or intensive care unit, required complete bed rest, and were cared for by professional staff. The nurses responsible for taking care of the patients in the high-risk group have rich clinical experience and hold a mid-level or above professional title. After the bleeding was controlled, especially during the peak bleeding periods (5:00–6:00, 17:00–24:00), the high-risk patients were highly vigilant and the nurses closely observed the patients’ conditions. If hematemesis occurred, the blood stains at the mouth and nose were immediately cleaned to prevent respiratory tract obstruction. If coma or drowsiness occurred, it may be due to hypoxia and appropriate oxygen was given. When the patient was discharged, their address and contact information were registered. Within half a year, the patient was followed up by phone once every 2 weeks, and once a month in the first 3 months. The post-discharge condition of the patient was evaluated and guidance was given for their out-of-hospital rehabilitation training.

#### Observation indicators

(1) The hemostatic time and hospital stay were compared between the two groups.

(2) The patients were followed up for half a year, and the number of patients with re-bleeding and death was recorded. Criteria for re-bleeding: increased incidence of hematemesis and black stool; Peripheral circulatory failure did not improve or worsen significantly; Red blood cell count, hemoglobin concentration, urea nitrogen continued to increase.

(3) The Glasgow-Blatchford score of patients was assessed by trained doctors who did not take part in the treatment before the nursing intervention in hospital and at the time of discharge.

(4) Total incidence of complications including pressure sores, lower limb vein thrombosis and infection was recorded and compared between the two groups.

(5) The psychological states of patients were measured by self-rating anxiety scale (SAS) and self-rating depression scale (SDS) (14). Both scales had 20 items, and the critical values were 50 points and 53 points respectively. The higher the score, the more serious the anxiety and depression.

(6) Exercise of self-care agency (ESCA) was used for assessing patients’ self-care ability (15), including health knowledge level (68 points), self-concept (32 points), self-care skills (48 points), and self-responsibility (24 points). The higher the score, the better the self-care ability.

(7) The Short Form-36 (SF-36) was used for assessing patients’ quality of life (16), including 8 dimensions such as physiological function, role physical, body pain, general health, vitality, social function, emotional function and mental health. The total score was 100 points. The higher the score, the better quality of life.

(8) The nursing satisfaction of patients was assessed by the Newcastle Satisfaction with Nursing Scale (NSNS) (17), with a full score of 100, which was divided into very satisfied (90∼100), satisfied (70∼89), as well as dissatisfied (<70). Nursing satisfaction = (very satisfied + satisfied) Number of cases/total cases × 100%.

### Statistical analysis

SPSS 25.0 software was adopted for statistical analysis. The statistical data were expressed as rate and χ^2^ test was used for comparison. Measurement data were expressed in (x ± s) and t test was adopted for comparison. *P* < 0.05 meant the difference was significant.

## Results

### There was no significant difference in general data of patients between the two groups

There was no significant difference in general data including sex, age and Child-Pugh grading of patients between the two groups (*P* > 0.05, Table 1).

**TABLE 1:** General data of patients in both groups.

Groups	Cases	Sex		Age (years)	Child-Pugh grading		
		Male	Female		A	B	C
Control group	40	20 (50.00)	20 (50.00)	52.24 ± 6.25	5 (12.50)	20 (50.00)	15 (37.50)
Study group	40	22 (55.00)	18 (45.00)	52.28 ± 6.31	6 (15.00)	21 (52.50)	13 (32.50)
t/χ^2^		0.20		0.02	0.25		
*P*		0.65		0.97	0.87		

### Graded nursing based on the Glasgow-Blatchford score significantly shortened the hemostatic time and hospital stay of patients with liver cirrhosis combined with AUGIB

**FIGURE 1:**
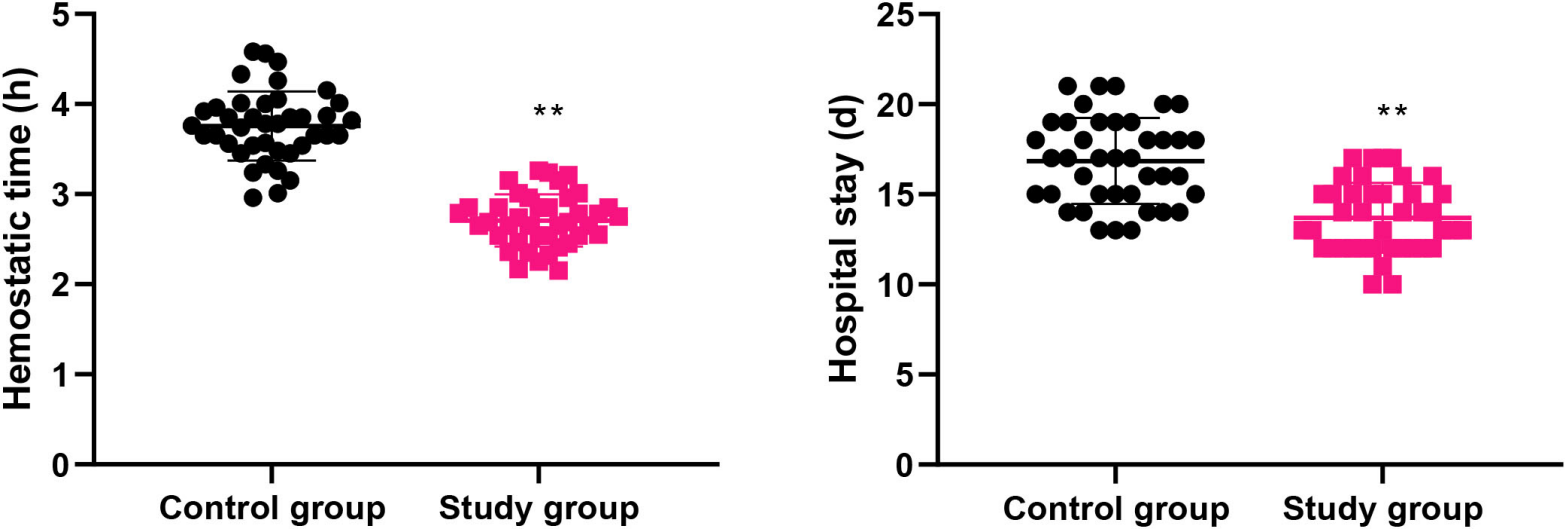
Hemostatic time and hospital stay in both groups. **P < 0.01.

The hemostatic time and hospital stay of the control group were (3.75 ± 0.38) h and (16.85 ± 2.38) d, respectively, while those in the study group were (2.70 ± 0.28) h and (13.70 ± 1.93) d, respectively. Compared to the control group, the study group had shorter hemostatic time and hospital stay (*P* < 0.01, Figure 1).

### Graded nursing based on the Glasgow-Blatchford score significantly reduced the rate of re-bleeding and death in patients with liver cirrhosis combined with AUGIB

The re-bleeding and death rate of the control group were 27.50% and 17.50%, respectively, while those of the study group were 7.50% and 2.00%, respectively. Relative to the control group, the study group had lower rate of re-bleeding and death (*P* < 0.05, Table 2).

**TABLE 2:** Re-bleeding and death in both groups.

Groups	Cases	Re-bleeding	Death
Control group	40	11 (27.50)	7 (17.50)
Study group	40	3 (7.50)	1 (2.00)
χ^2^		5.54	5.00
*P*		0.01	0.02

### Graded nursing based on the Glasgow-Blatchford score significantly reduced the Glasgow-Blatchford score in patients with liver cirrhosis combined with AUGIB

Before intervention, the Glasgow-Blatchford score in the control group was (12.48 ± 1.25) points, and that in the study group was (12.50 ± 1.26) points. At discharge, the Glasgow-Blatchford score in the control group was (9.06 ± 0.91) points, and that in the study group was (6.32 ± 0.63) points.

**FIGURE 2:**
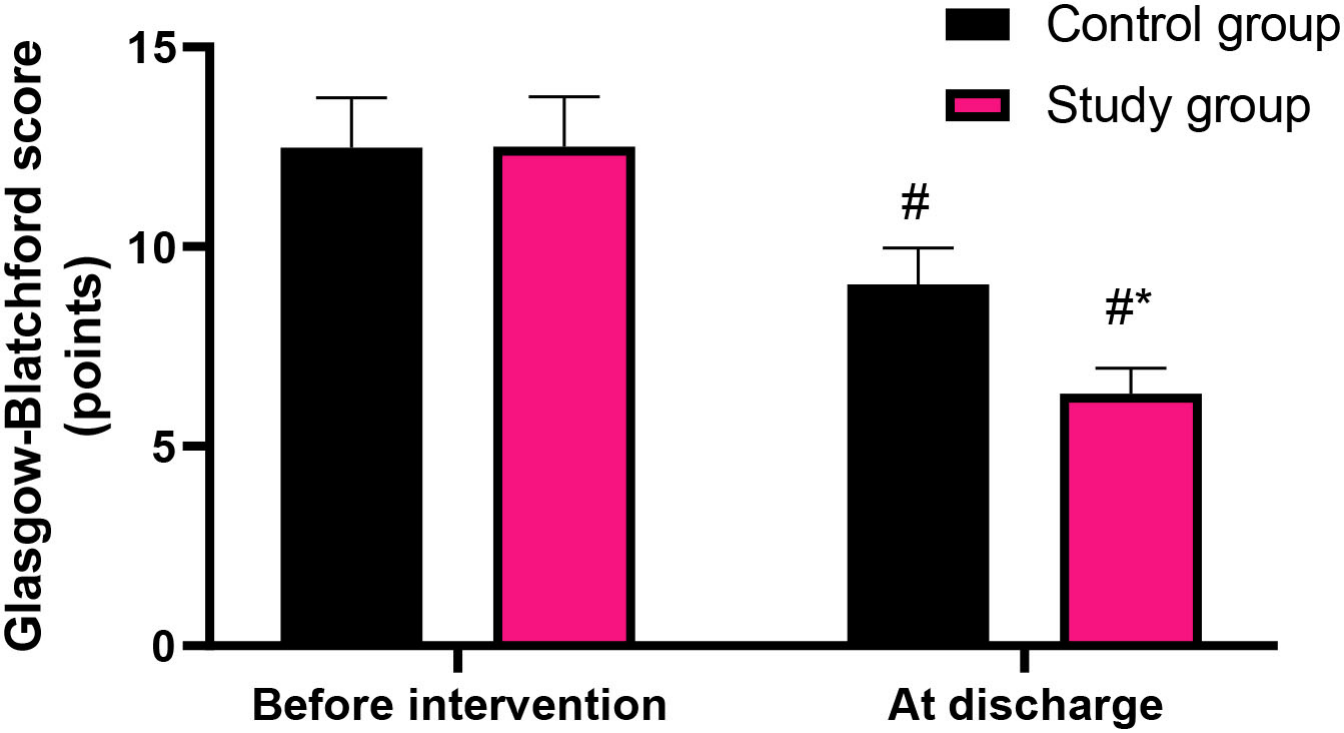
Glasgow-Blatchford score in both groups. ^#^*P* < 0.05, vs. before intervention; *P < 0.05, vs. control group.

Before intervention, no difference was seen in Glasgow-Blatchford score between both groups (*P* > 0.05). At discharge, Glasgow-Blatchford score was declined in both groups (*P* < 0.05). Relative to the control group, the study group had lower Glasgow-Blatchford score at discharge (*P* < 0.05, Figure 2).

### Graded nursing based on the Glasgow-Blatchford score significantly reduced the incidence of complications in patients with liver cirrhosis combined with AUGIB

In the control group, there were 3 cases of pressure sores, 5 cases of lower limb vein thrombosis and 3 cases of infection. The total incidence rate was 27.50%. In the study group, there was 1 case of pressure sores and 1 case of infection. The total incidence rate was 5.00%. Compared to the control group, the study group had lower incidence of complications (*P* < 0.01, Table 3).

**TABLE 3:** Incidence of complications in both groups.

Groups	Cases	Pressure sores	Lower limb vein thrombosis	Infection	Total incidence rate
Control group	40	3 (7.50)	5 (12.50)	3 (7.50)	11 (27.50)
Study group	40	1 (2.50)	0 (0.00)	1 (2.50)	2 (5.00)
χ^2^					9.93
*P*					<0.01

### Graded nursing based on the Glasgow-Blatchford score significantly alleviated the anxiety and depression in patients with liver cirrhosis combined with AUGIB

Before intervention, the SAS and SDS scores in the control group were (52.65 ± 5.27) points and (45.85 ± 4.59) points, respectively, and those in the study group were (52.68 ± 5.31) points and (45.91 ± 4.62) points, respectively.

After intervention, the SAS and SDS scores in the control group were (45.16 ± 4.52) points and (40.26 ± 4.03) points, respectively, and those in the study group were (41.12 ± 4.13) points and (36.45 ± 3.65) points, respectively.

**FIGURE 3:**
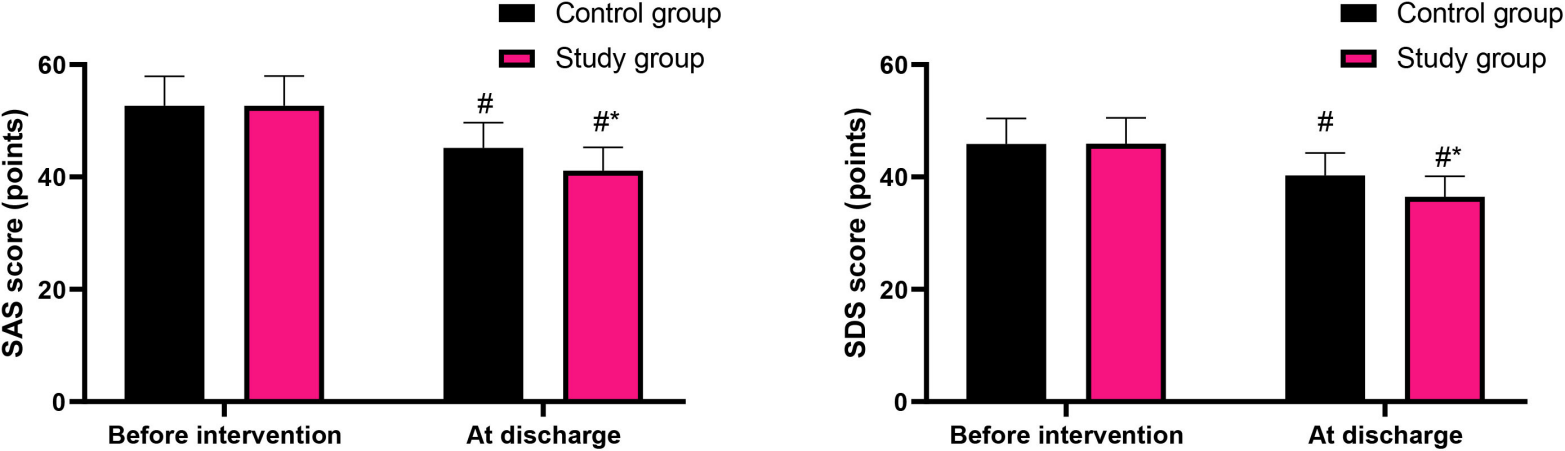
Psychological states of patients in both groups. ^#^*P* < 0.05, vs. before intervention; *P < 0.05, vs. control group.

Before intervention, no difference was seen in SAS and SDS scores between both groups (*P* > 0.05). At discharge, SAS and SDS scores were declined in both groups (*P* < 0.05). Relative to the control group, the study group had lower SAS and SDS scores at discharge (*P* < 0.05, Figure 3).

### Graded nursing based on the Glasgow-Blatchford score significantly promoted the self-care ability of patients with liver cirrhosis combined with AUGIB

Before intervention, the ESCA scores in the aspects of health knowledge level, self-concept, self-care skills, and self-responsibility in the control group were (25.75 ± 2.58) points, (14.49 ± 1.52) points, (19.68 ± 2.03) points and (13.89 ± 1.42) points, respectively, and those in the study group were (25.71 ± 2.57) points, (14.43 ± 1.48) points, (19.63 ± 1.95) points and (13.81 ± 1.39) points, respectively.

After intervention, the ESCA scores in the aspects of health knowledge level, self-concept, self-care skills, and self-responsibility in the control group were (34.59 ± 3.52) points, (20.65 ± 2.06) points, (25.69 ± 2.61) points and (18.26 ± 1.83) points, respectively, and those in the study group were (41.23 ± 4.15) points, (26.78 ± 2.68) points, (33.45 ± 3.36) points and (22.54 ± 2.26) points, respectively.

**FIGURE 4:**
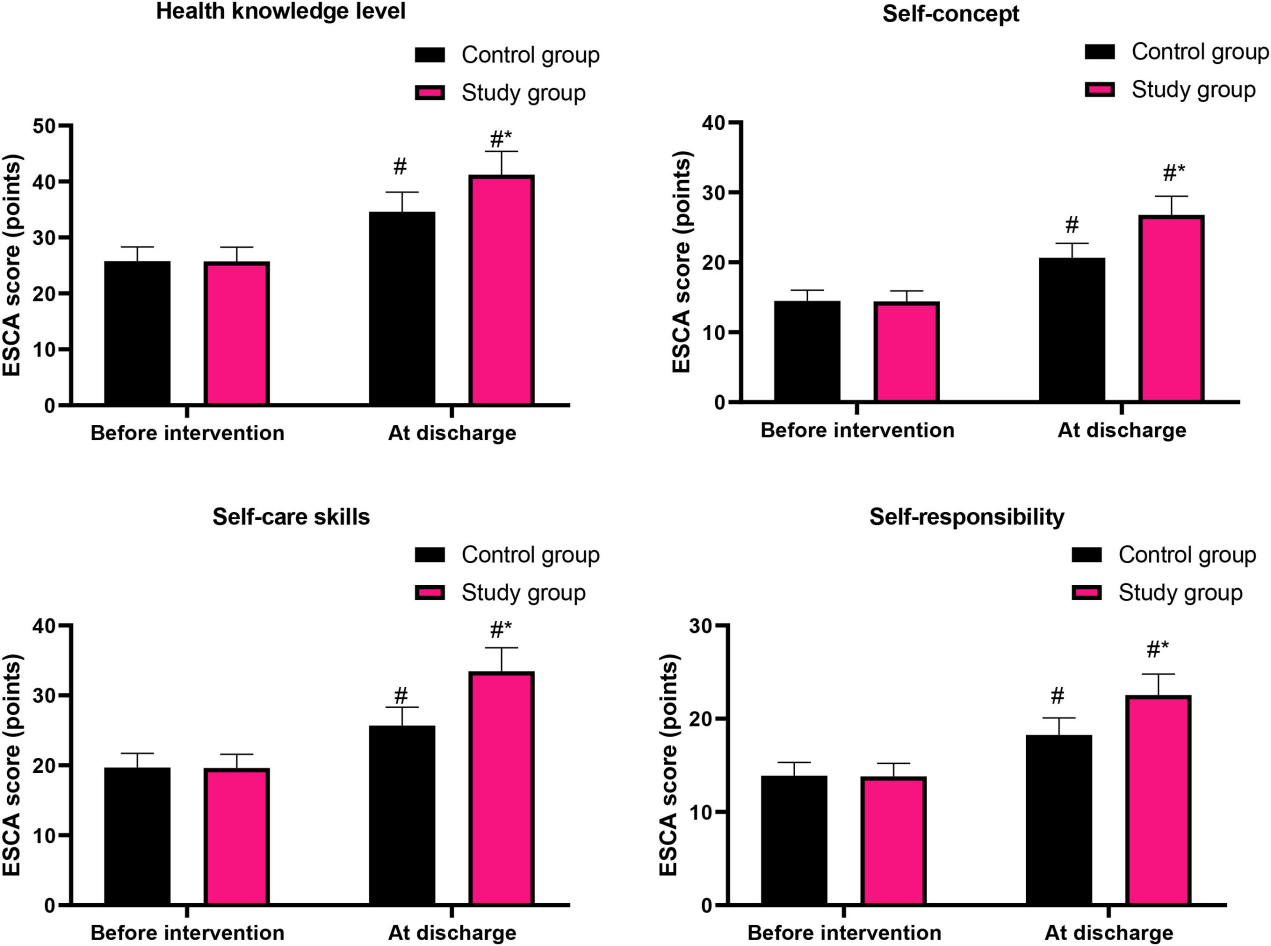
Patients’ self-care ability in both groups. ^#^*P* < 0.05, vs. before intervention; *P < 0.05, vs. control group.

Before intervention, no difference was seen in the ESCA scores between both groups (*P* > 0.05). At discharge, ESCA scores were elevated in both groups (*P* < 0.05). Relative to the control group, the study group had higher ESCA scores at discharge (*P* < 0.05, Figure 4).

### Graded nursing based on the Glasgow-Blatchford score significantly improved the quality of life in patients with liver cirrhosis combined with AUGIB

Before intervention, the SF-36 scores in the aspects of physiological function, role physical, body pain, general health, vitality, social function, emotional function and mental health in the control group were (65.96 ± 6.62) points, (66.52 ± 6.65) points, (68.52 ± 6.87) points, (70.26 ± 7.03) points, (71.62 ± 7.18) points, (70.69 ± 7.08) points, (71.23 ± 7.13) points and (70.65 ± 7.07) points, respectively, and those in the study group were (65.91 ± 6.59) points, (76.85 ± 7.68) points, (68.48 ± 6.82) points, (70.21 ± 7.02) points, (71.59 ± 7.15) points, (70.59 ± 7.05) points, (71.16 ± 7.15) points and (70.41 ± 7.04) points, respectively.

After intervention, the SF-36 scores in the aspects of physiological function, role physical, body pain, general health, vitality, social function, emotional function and mental health in the control group were (75.69 ± 7.58) points, (66.48 ± 6.59) points, (77.36 ± 7.74) points, (78.62 ± 7.82) points, (77.26 ± 7.78) points, (76.25 ± 7.63) points, (80.65 ± 8.07) points and (76.98 ± 7.69) points, respectively, and those in the study group were (83.69 ± 8.42) points, (85.21 ± 8.41) points, (86.32 ± 8.14) points, (85.47 ± 8.54) points, (84.69 ± 8.53) points, (85.65 ± 8.57) points, (85.69 ± 8.51) points and (84.96 ± 8.49) points, respectively.

**FIGURE 5:**
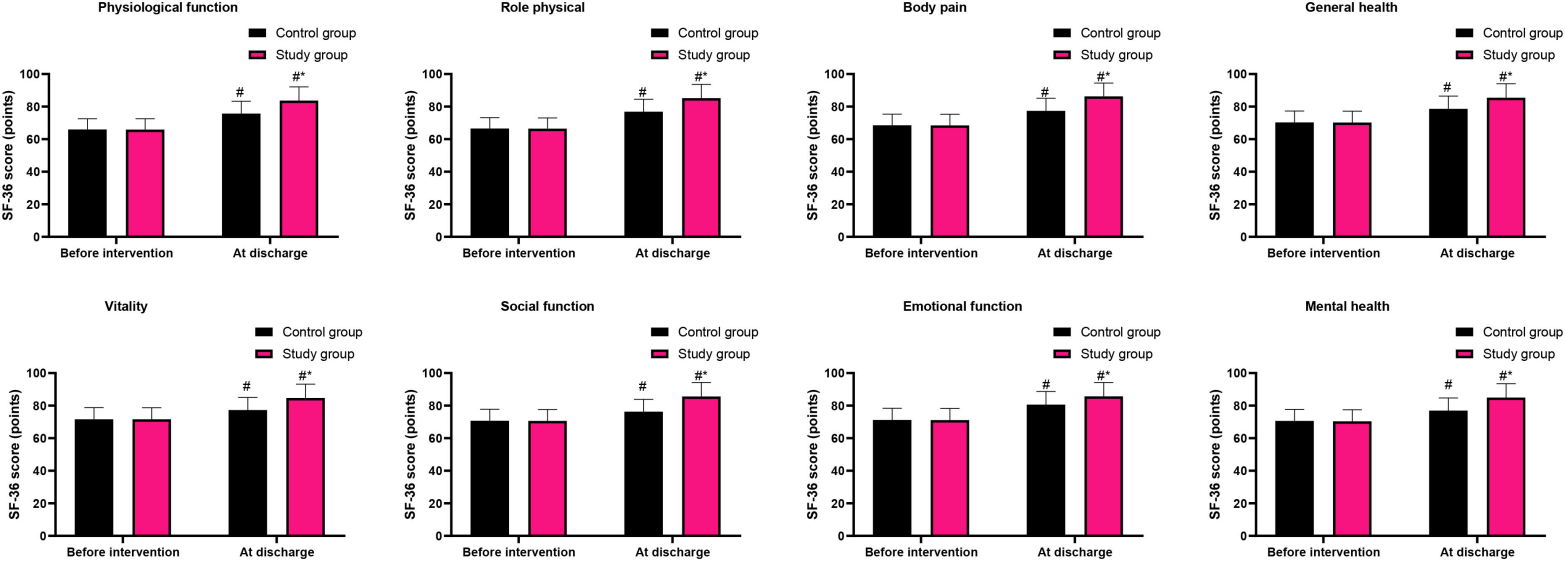
Quality of life in both groups. ^#^*P* < 0.05, vs. before intervention; *P < 0.05, vs. control group.

Before intervention, no difference was seen in SF-36 scores between both groups (*P* > 0.05). At discharge, SF-36 scores were elevated in both groups (*P* < 0.05). Relative to the control group, the study group had higher SF-36 scores at discharge (*P* < 0.05, Figure 5).

### Graded nursing based on the Glasgow-Blatchford score significantly increased the nursing satisfaction of patients with liver cirrhosis combined with AUGIB

In the control group, there were 15 cases were very satisfied with the nursing, 16 cases were satisfied with the nursing, and 9 cases were dissatisfied with the nursing. The total satisfaction rate was 77.50%. In the study group, there were 20 cases were very satisfied with the nursing, 19 cases were satisfied with the nursing, and 1 case was dissatisfied with the nursing. The total satisfaction rate was 97.50%. Compared to the control group, the study group had higher nursing satisfaction (*P* < 0.01, Table 4).

**TABLE 4:** Nursing satisfaction of patients in both groups.

Groups	Cases	Very satisfied	Satisfied	Dissatisfied	Total satisfaction rate
Control group	40	15 (37.50)	16 (40.00)	9 (22.50)	31 (77.50)
Study group	40	20 (50.00)	19 (47.50)	1 (2.50)	39 (97.50)
χ^2^					7.31
*P*					<0.01

## Discussion

Acute upper gastrointestinal bleeding is the most serious complication of liver cirrhosis and an important cause of death for patients with liver cirrhosis (18). For patients with liver cirrhosis complicated with AUGIB, traditional nursing mainly relies on the patient’s clinical symptoms to predict the severity and likelihood of gastrointestinal bleeding, and then intervenes based on clinical experience combined with the patient’s clinical symptoms. This approach is relatively subjective and vague, resulting in unsatisfactory nursing intervention effects and hemostasis efficiency (19). Therefore, the current research focuses on further exploring the best nursing plan and improving the hemostasis efficiency of patients with liver cirrhosis complicated with AUGIB.

The Glasgow-Blatchford score is a commonly utilized systematic assessment method, often employed for evaluating various cases of AUGIB (20). In our study, the Glasgow-Blatchford score was adopted to assess the risk level of patients with liver cirrhosis complicated with AUGIB. According to the evaluation results, patients were classified into low-risk, medium-risk and high-risk groups, so that different intervention measures could be implemented, thereby rationally allocating scarce nursing resources and ensuring that patients with a high risk of severe bleeding receive adequate care while those with a mild risk of bleeding receive sufficient care.

In our study, the results suggested that compared with the control group, the study group had shorter hemostatic time and hospital stay. This is because that the Glasgow-Blatchford score, as a simple and efficient tool, is mainly used to evaluate AUGIB patients by observing blood pressure, heart rate, stool color, blood urea and hemoglobin and other indicators (21). Based on this, the risk assessment and graded nursing for patients with liver cirrhosis complicated with AUGIB can enhance nurses’ early warning awareness, predictive nursing ability and clinical decision-making ability, thereby shortening the time of hemostasis and promoting the recovery of disease symptoms.

Besides, the results of our study indicated that compared with the control group, the study group had lower re-bleeding rate, lower death rate and lower incidence of complications. Besides, compared with the control group, the study group had lower Glasgow-Blatchford score at discharge. This is because that graded nursing based on the Glasgow-Blatchford score is highly targeted, especially for patients with medium-risk and high-risk. It made special arrangements in terms of the selection of nurses and the selection of wards. In this way, even if the patient experiences re-bleeding, it can be promptly treated to save the patient’s life, thereby reducing the number of deaths, the re-bleeding rate, and the incidence of complications (22).

Moreover, our study indicated that relative to the control group, the study group had lower SAS and SDS scores at discharge. These results indicated that graded nursing based on the Glasgow-Blatchford score could alleviate the degree of anxiety and depression in cirrhosis patients combined with AUGIB. In addition, our study showed that relative to the control group, the study group had higher ESCA and SF-36 scores at discharge. At the same time, relative to the control group, the study group had higher nursing satisfaction. These results indicated that graded nursing based on the Glasgow-Blatchford score could promote the self-care ability, quality of life and nursing satisfaction of patients with cirrhosis combined with AUGIB. This is because that the graded nursing based on the Glasgow-Blatchford score can reasonably classify patients according to their conditions, plan the location of wards, provide care as needed for patients, fully meet their psychological and physiological needs, promptly control bleeding, reduce the impact of the disease on patients’ emotions, thereby alleviating their anxiety and depression, improving their self-care ability and quality of life. Consistently, Chen et al. suggested that graded nursing based on the risk early warning concept could reduce the occurrence of complications, promote activities of daily living and motor function and quality of life, relieve negative emotions, and improve patient satisfaction of long-term bedridden patients (23).

This study focuses on the application of graded nursing based on the Glasgow-Blatchford score in patients with liver cirrhosis and AUGIB, which holds extremely significant practical importance. From a clinical perspective, liver cirrhosis combined with AUGIB is a critical and complicated condition with numerous complications. The traditional nursing model often lacks specificity and systematicness, making it difficult to meet the individualized needs of patients with varying degrees of severity. However, the grading nursing model proposed in this study based on the Glasgow-Blatchford score can classify patients according to their score results into different risk levels and formulate corresponding nursing plans. This precise nursing model enables nurses to more reasonably allocate nursing resources, devote more energy and attention to patients with more severe conditions, and promptly take effective nursing measures, thereby reducing the re-bleeding rate and the incidence of adverse reactions. Meanwhile, the graded nursing model also places emphasis on providing psychological support and health education to patients. Patients with liver cirrhosis combined with AUGIB often experience anxiety and depression due to the sudden and severe nature of their condition. These emotions not only affect the patients’ compliance with treatment but may also further aggravate the condition. By providing psychological counseling and emotional support to patients during the nursing process, helping them build confidence in overcoming the disease, and alleviating their anxiety and depression, it is beneficial for their physical and mental recovery. In addition, strengthening health education for patients and enhancing their self-care ability enable them to better manage themselves after discharge, follow medical advice, have a balanced diet, maintain a regular schedule, and thereby reduce the recurrence of the disease and improve their quality of life. From the perspectives of patients and society, the results of this study can provide patients with higher-quality and more efficient nursing services, enhance patients’ satisfaction with care, and improve their medical experience. At the same time, it can reduce the re-bleeding rate and the incidence of adverse reactions, shorten the hospitalization time and medical expenses for patients, and alleviate the economic burden on their families. Moreover, it can improve patients’ self-care ability and quality of life, helping them better reintegrate into society, reducing social functional loss caused by diseases, and having positive significance for the development and stability of society.

This research has multiple positive impacts on the development of medicine. In the field of nursing, this study provides a new model and method for the care of patients with liver cirrhosis complicated by AUGIB. The traditional nursing model is mainly based on doctors’ orders and experience, lacking scientific and systematic assessment tools and personalized care plans. However, the graded nursing model based on the Glasgow-Blatchford score combines risk assessment with nursing intervention, making the nursing work more precise and effective. The promotion and application of this model are expected to improve the service level and quality of the entire nursing industry, and promote the development of the nursing discipline toward a more specialized and scientific direction.

In terms of clinical decision-making, the Glasgow-Blatchford score serves as a simple and practical assessment tool, enabling doctors to quickly and accurately evaluate the severity and prognosis of patients, and providing important basis for the formulation of treatment plans. This study further verified the application value of this score in graded nursing, providing more scientific references for clinical doctors in their nursing decisions. At the same time, the research results also offer insights and inspirations for the risk assessment and nursing intervention of other similar diseases, contributing to the advancement of clinical medicine toward a more precise and personalized direction.

In the field of medical research, this study provides a foundation and reference for subsequent related research. By conducting an in-depth exploration of the effect of graded nursing based on the Glasgow-Blatchford score in patients with liver cirrhosis and AUGIB, it reveals the impact of this nursing model on the re-bleeding rate, incidence of adverse reactions, psychological state, self-care ability, and quality of life of the patients. This provides directions and ideas for further research on the long-term effect, mechanism of action, and combined application with other treatment methods of this nursing model.

This research has several novel aspects. In terms of research perspective, although there have been some studies on the nursing care of patients with liver cirrhosis combined with acute upper gastrointestinal bleeding, most of them focused on the evaluation of the effect of a single nursing measure, lacking systematic research from the perspective of an overall nursing model. This study is the first to introduce the Glasgow-Blatchford score into graded nursing, and has constructed a personalized nursing model based on risk assessment. It comprehensively evaluates the nursing effect from multiple dimensions, providing a new perspective for research in this field. In terms of research methods, this study adopted the design of a randomized controlled trial. Patients were randomly divided into the control group and the experimental group, and were respectively given routine care and graded care based on the Glasgow-Blatchford score. By comparing the various indicators of the two groups of patients, the effect of graded care was objectively and accurately evaluated. This rigorous research method can effectively control the interference of confounding factors and improve the reliability and credibility of the research results. At the same time, this study also used various assessment tools to comprehensively and systematically assess the psychological state, self-care ability, and quality of life of the patients, making the research results more comprehensive and in-depth.

However, there are many shortcomings in this study, such as single source of cases and small number of cases, which may result in the bias of the research results. In the future, multi-center large-sample studies should be implemented to support the results of this study and provide more objective guidance basis for clinical nursing of patients with cirrhosis combined with AUGIB.

## Conclusion

Graded nursing based on the Glasgow-Blatchford score can reduce the re-bleeding rate and incidence of adverse reactions, alleviate the anxiety and depression and enhance the self-care ability, quality of life and nursing satisfaction of patients with liver cirrhosis combined with AUGIB.

## Data Availability

The original contributions presented in this study are included in this article/supplementary material, further inquiries can be directed to the corresponding author.
